# Bouba/Kiki in Touch: Associations Between Tactile Perceptual Qualities and Japanese Phonemes

**DOI:** 10.3389/fpsyg.2018.00295

**Published:** 2018-03-12

**Authors:** Maki Sakamoto, Junji Watanabe

**Affiliations:** ^1^Department of Informatics, The University of Electro-Communications, Tokyo, Japan; ^2^NTT Communication Science Laboratories, Nippon Telegraph and Telephone Corporation, Kanagawa, Japan

**Keywords:** cross-modal association, touch, sound-symbolic word, material texture, perception

## Abstract

Several studies have shown cross-modal associations between sounds and vision or gustation by asking participants to match pre-defined sound-symbolic words (SSWs), such as “bouba” or “kiki,” with visual or gustatory materials. Here, we conducted an explorative study on cross-modal associations of tactile sensations using spontaneous production of Japanese SSWs and semantic ratings. The Japanese language was selected, because it has a large number of SSWs that can represent a wide range of tactile perceptual spaces with fine resolution, and it shows strong associations between sound and touch. In the experiment, we used 120 everyday materials to cover basic material categories that could be associated with fundamental dimensions of tactile perception. Upon contact with these materials, participants expressed their tactile sensations by using Japanese SSWs, and at the same time, evaluated the tactile sensations by semantic differential scales using adjective pairs. Thanks to the variety of testing materials, we were able to demonstrate the existence of systematic associations between sounds and tactile fundamental perceptual dimensions in a more detailed and comprehensive way than ever done so before. In particular, we found that for vowels, positive tactile ratings were associated with the back vowel (/u/), while negative ratings were associated with the front vowels (/i/ and /e/). The central vowels (/o/ and /a/) were mainly associated with rough, hard, and dry feelings. Consonants were categorized based on vocal features and articulation. The category of the voiced consonants (e.g., /dz/ and /g/) corresponded to feelings of roughness, while that of voiceless consonants (e.g., /ʦ/, and /s/) corresponded to feelings of smoothness. The categories of the bilabial plosive (/p/ and /b/) and voiced alveolar nasal (/n/) consonants were mainly related to soft, sticky and wet feelings, while that of voiceless alveolar affricate (/ʦ/) and voiceless velar plosive (/k/) consonants were related to hard, slippery and dry feelings.

## Introduction

Contrary to traditional linguistic thought that relationships between speech sounds and word meanings are arbitrary, recent studies have suggested that iconicity (aspects of form resembling aspects of meaning) rather plays some role in structuring vocabulary (for a review, see Dingemanse et al., 2015). Some studies have studied phonological iconicity, i.e., “sound symbolism,” as contributing to the understanding of the development of language abilities (e.g., Westbury, 2004; Maurer et al., 2006; Asano et al., 2015) and language evolution (Ohala, 1997; Ramachandran and Hubbard, 2001). Over the decades, many studies have demonstrated the existence of sound symbolism (e.g., Jespersen, 1922; Köhler, 1929; Sapir, 1929; for early studies) in languages worldwide (e.g., Brown et al., 1955; Davis, 1961; Emeneau, 1969; Klank et al., 1971; Hinton et al., 1994; Nuckrolls, 1999; Voeltz and Kilian-Hatz, 2001; Enfield, 2005; Kovic et al., 2010; Bremner et al., 2013; Schmidtke et al., 2014). Even in Indo-European languages, such as English, clear sound symbolism is evident (Bloomfield, 1933; Bolinger, 1950; Crystal, 1995). For example, roughly half of the English words starting with “gl-” imply something visual and bright, such as glance, glare, gleam, glimmer, glamor, glass, glaze, glimpse, glint, glisten, glitter, globe, glossy, and glow (Crystal, 1995).

Landmark studies have found sound symbolism in words referring to visual shapes, such as mal vs. mil and bouba vs. kiki for round vs. sharp shapes, respectively (Sapir, 1929; Ramachandran and Hubbard, 2001, respectively). Sound symbolism in vision has been discussed in terms of the process by which speakers link phonetic features with meanings (D'Onofrio, 2013). For instance, speakers associate nonsense words articulated with rounded lips, such as “bouba” with round shapes, and nonsense words articulated without rounded lips, such as “kiki” with sharp shapes. In addition, the number of studies investigating sound symbolism in gustation is growing (e.g., Gallace et al., 2011; Sakamoto and Watanabe, 2016). Gallace et al. (2011), for example, showed that salt and vinegar crisps such as potato chips tend to be rated as “kiki” than cheddar cheese yogurt, or jam, and chocolate with mint chips and crisps also tend to be associated with “kiki” than regular chocolate. However, compared with vision and gustation, sound symbolism in tactile modality has received little attention. Although the existence of sound symbolism in touch has been suggested (Dingemanse, 2012; Dingemanse and Majid, 2012; Watanabe et al., 2012) and tested using pre-defined sounds (Fryer et al., 2014; Etzi et al., 2016), the associations have not been demonstrated in a comprehensive manner.

In this study, we explored sound symbolism in touch, especially the association between phonemes of Japanese sound-symbolic words (SSWs) expressing tactile sensations and evaluations of tactile materials. Japanese was our focus because Japanese, compared with other languages, has a large vocabulary of tactile SSWs (Japanese ideophones are called “onomatopoeia” in Japanese) and all Japanese phonemes are systematically associated with sound symbolic meanings (Hamano, 1998).

Most studies on tactile perception have used semantic differential scales of multiple adjective pairs (Okamoto et al., 2013), and have explored the fundamental dimensions of tactile perceptual space (e.g., rough/smooth, hard/soft, slippery/sticky, and warm/cold). Recently, Sakamoto and Watanabe (2017) used SSWs to collect tactile materials in a comprehensive manner, and investigated basic dimensions of tactile perceptions using semantic differential scales of adjectives. Unlike previous studies, the present study investigated sound symbolism of tactile SSWs because the SSWs classify perceptual space with finer resolution than adjectives and the sounds of Japanese SSWs are closely related to perceptual qualities. In fact, in one experiment, the variety of words between the two types of expressions (SSWs or adjective) was examined; participants touched 40 materials and expressed their sensations using SSWs or adjectives (Sakamoto and Watanabe, 2013). The results showed that 279 types of SSWs in 1,200 trials (40 materials × 30 participants) were obtained, while 124 types of adjective words (less than half of SSWs) were obtained. Furthermore, most Japanese SSWs expressing tactile sensations carry a two-syllable repetition form (C_1_V_1_C_2_V_2_ − C_1_V_1_C_2_V_2_, where C and V indicate consonant and vowel, respectively, e.g., “sara-sara”), and sounds of the first syllable (C_1_V_1_, e.g., “sa” for “sara-sara”) are strongly associated with evaluations of tactile sensations (Hamano, 1998). For instance, “sara-sara” and “zara-zara,” which only differ in the first syllable of the repetition unit, denote totally different tactile sensations. While the former is used for expressing smooth and pleasant touch, the latter is used for expressing rough and unpleasant touch. In contrast, although “beta-beta” and “beto-beto” only differ in the second syllable of their repetition unit, these SSWs express almost the same tactile sensation, namely stickiness. Therefore, we expect the possibility of the first syllable of Japanese SSWs acting as a guide for systematically exploring touch-sound correspondence.

In the experiment, while participants touched materials associated with fundamental tactile properties, they expressed their tactile sensations using Japanese SSWs. They also evaluated the sensations according to semantic differential scales of adjective pairs. We obtained a vast number of combinations for the first syllable of Japanese SSWs and evaluations of tactile sensations, and then examined features of sound symbolism in touch in terms of voiced/voiceless, location of articulation, and manner of articulation.

## Materials and methods

### Ethics statement

This study was approved by the ethics committee of The University of Electro-Communications, Tokyo, Japan. The study adhered to the tenets of the Declaration of Helsinki. All participants provided written informed consent prior to participation in the experiments. Documents about the experimental procedures and written informed consent forms were presented to the ethics committee.

### Participants

Fifteen native speakers of Japanese (10 men and five women, aged between 19 and 26 years old) volunteered to participate in the experiments without knowledge of the experiments' purposes. They had no abnormalities in either their verbal ability or any particular skills with respect to touch. They visited a laboratory at The University of Electro-Communications for 1 day to participate in trials.

### Stimuli

The collection of material samples followed the same procedure as the samples used in our previous study (Sakamoto and Watanabe, 2017), where we investigated fundamental perceptual dimensions in touch (see the detailed procedure of selection of material samples, and the photos of selected materials here: http://journal.frontiersin.org/article/10.3389/fpsyg.2017.00569/full). Sakamoto and Watanabe (2017) selected 238 SSWs, whose first syllables included all Japanese consonants except /l/ (/l/ is not used as a first syllable in existing SSWs expressing touch). Then they collected 120 material samples with each of the 238 SSWs expressing at least one of the material properties of the 120 materials. The 120 types of tactile material samples included glass, papers, stones, sand, metals, rubbers, woods, clay and plastics (see the entire list of materials here: https://www.frontiersin.org/articles/10.3389/fpsyg.2017.00569/full#supplementary-material). The samples were cut to 6 × 6 cm, and stacked in layers to 2 mm thickness. The stones and sand were stored loose in a container.

### Procedure

Each participant sat in front of a box that had an 8 × 10 cm hole (hereon referred to as the material box). They put the index finger of their dominant hand through the hole of the material box to touch a material; they could not see the material while they were touching it. One trial comprised a describing period and a rating period. In advance, participants were instructed on SSWs with examples unrelated to touch, and were informed of the procedure for expressing their tactile feelings using a sound-symbolic expression and adjectives during the describing and rating periods, respectively. During the 30 s describing period, while touching one of the 120 materials, participants spontaneously reported a sound-symbolic expression to describe the tactile feeling without seeing any examples. During the rating period, while touching the same material, the participants evaluated the sensations of the touched material in terms of a seven-point scale for eight adjective pairs (comfort—discomfort, bumpy—flat, rough—smooth, hard—soft, non-elastic—elastic, slippery—sticky, dry—moist, and warm—cold). The adjectives were selected by referring to a review paper on research investigating tactile perceptual dimensions (Okamoto et al., 2013). The selected adjectives were comfort/discomfort rating and fundamental dimensions frequently used in previous tactile studies. No limit was imposed on the answering time for the rating period.

Note that during the describing period, participants could generate a novel sound-symbolic expression. The SSWs in Japanese are easily produced by combining parts of existing vocabulary (for example, “mofu-mofu,” a newly produced sound-symbolic expression, is a combination of “moko-moko” and “fuwa-fuwa”). By using the spontaneously produced expression, we could include novel expressions that were not included in pre-defined testing vocabulary of the research design. Thus, we could directly investigate relationships between sounds and tactile perceptual experiences.

While the participants described and rated the tactile sensations, participants were free to run their fingers along, and push against the surface of the materials to explore the materials' various properties. Each participant performed 120 trials (a single trial for each of the 120 materials). For each trial, the experimenter placed one material in the material box, and the participant touched it and responded, then the material was replaced with another after the participant responded. Material samples were randomly presented.

## Results

For each adjective pair, we obtained 1,800 combinations of sound-symbolic expressions and evaluations (120 materials × 15 participants). In 87.1% of all trials (1,566 cases), the sound-symbolic expressions were in two-syllable repetition form. Because the Japanese linguistic form also has sound-symbolic meaning (Hamano, 1998), we used the first syllables of these 1,566 instances to examine sound symbolism in touch. Approximately 20% of the 1,566 cases were novel sound-symbolic expressions. We obtained 22 first syllables that occurred more than 16 times (1% of 1,566), and decided to use the 22 syllables to explore sound symbolism in touch. Table 1 shows the evaluations of 22 syllables for each adjective pair. The values, which were significantly different from the average of 1,566 cases for each scale (*t*-test, *p* < 0.05), and whose difference in absolute value from the average were larger than 0.5, are shown. Positive values are in orange (upper adjective in the first row), while negative values are in light blue. For example, in the first colored column (comfort/discomfort), an average of 0.11 was obtained (i.e., a little comfort), with values in the orange and light blue areas relating to comfort and discomfort, respectively. For all adjective pairs, we found several types of associations between the first syllables and tactile qualities.

**Table 1:** List of first syllables that were obtained more than 16 times (1% of all responses).

	***N* = 1,397/1,566**	**Comfort Discomfort (0.11)**	**Bumpy Flat (−0.12)**	**Rough Smooth (−0.06)**	**Hard Soft (−0.18)**	**Non-elastic Elastic (0.05)**	**Slippery Sticky (0.25)**	**Dry Moist (0.43)**	**Warm Cold (−0.41)**
/ʦ/+/u/	149	0.92	−1.87	−1.70	1.34	1.21	1.89	1.01	−1.18
/s/+/u/	94	1.13	−1.67	−1.78		0.87	1.70		
/s/+/a/	130	0.68		−0.68			1.14	1.08	
/p/+/a/	22								
/p/+/o/	29								
/k/+/a/	52				1.23	0.79	0.98	1.77	
/ɸ/+/u/	118	1.30			−1.73	−1.16			0.91
/m/+/o/	32	1.19	1.06	1.00					1.00
/g/+/a/	50		1.40	1.28	0.84			1.00	
/d/+/a/	24		1.38	1.38	0.96			1.33	
/b/+/o/	63	−0.56	1.95	1.27	0.87	0.78			
/dz/+/a/	195	−0.49	0.87	1.52	0.48	0.88		1.24	
/g/+/o/	52		1.33	0.92	1.27	1.00		1.10	
/tɕ/+/i/	23	−0.87	1.35	2.52				1.43	
/b/+/u/	27					−1.33	−0.74	−1.00	
/g/+/u/	32		0.63		−1.44	−1.44	−0.66	−1.06	
/p/+/u/	57	0.63		−0.98	−2.05	−2.21		−1.49	
/ɕ/+/i/	18							−0.89	
/p/+/i/	22		−1.00					−1.91	−1.82
/b/+/e/	101	−1.41	−0.96		−1.50	−1.00	−2.29	−1.35	
/n/+/e/	26	−1.19		−0.88	−1.73	−1.04	−2.58	−1.81	
/p/+/e/	81	−0.41	−1.23	−0.64	−1.00	−0.65	−1.49	−1.00	

*The values, which are significantly different from the average of 1566 cases for each scale (t-test, p < 0.05), and whose difference in absolute value from the average are larger than 0.5, are shown. Positive values are in orange (upper adjective in the first row), while negative values are in light blue*.

**Figure 1:**
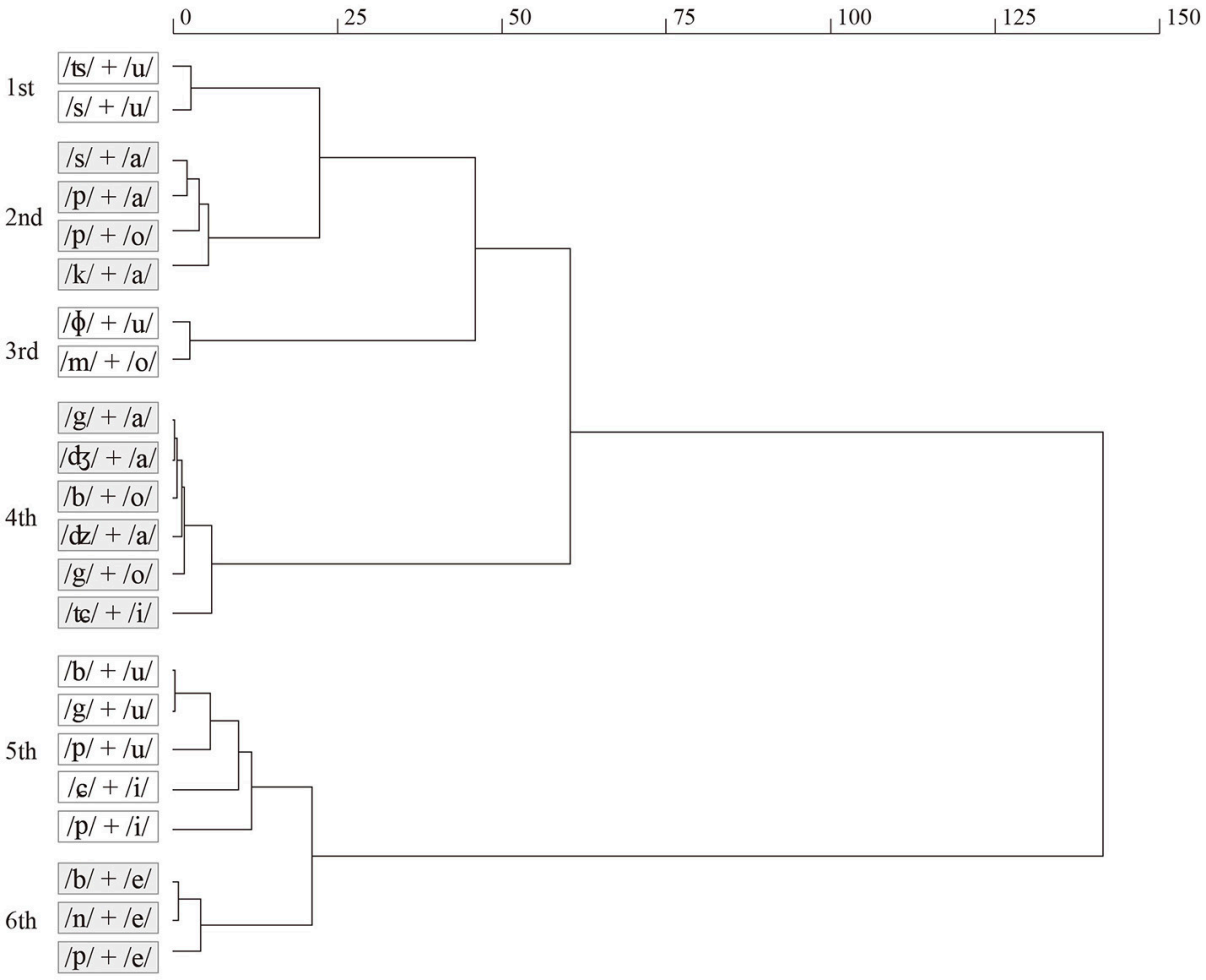
Result of cluster analysis for 22 syllables. The level of cluster was decided until the square distance reacged one-tenth that of the first bifurcation.

We also performed hierarchical cluster analysis (Ward's clustering algorithm) using mean values of the 22 syllables in terms of eight adjective pairs. Figure 1 shows the result of the cluster analysis. The first cluster (/ʦ/+/u/ and /s/+/u/) was related to the comfort, flat, smooth, hard, non-elastic, slippery, dry, and cold evaluations (see also Table 1). These phonemes were obtained when touching materials such as glass, and metals. The second cluster (/s/+/a/, /p/+/a/, /p/+/o/, and /k/+/a/) was related to the slippery and dry evaluations, obtained by materials such as paper, and plastics. The third cluster (/ɸ/+/u/ and /m/+/o/) was related to the comfort, bumpy, rough, soft, elastic, and warm evaluations, obtained by materials such as soft fabrics. The fourth cluster (/g/+/a/, /dʒ/+/a/, /b/+/o/, /dz/+/a/, /g/+/o/, and /tɕ/+/i/) was related to the discomfort, bumpy, rough, hard, non-elastic, and dry evaluations, obtained by materials such as woods, stones, and sand paper. The fifth cluster (/b/+/u/, /g/+/u/, /p/+/u/, /ɕ/+/i/ and /p/+/i/) was related to the elastic and wet evaluations, obtained by materials such as rubber. The sixth cluster (/b/+/e/, /n/+/e/, and /p/+/e/) was related to the discomfort, flat, smooth, soft, elastic, sticky, and wet evaluations obtained by materials such as slime.

Tables 2–**9** were constructed in terms of articulation of the Japanese language. In the tables, the syllables are first classified into “voiced” and “voiceless” panels, i.e., with/without vibration of the vocal cords, because this feature has been reported to be critical in the impressions of SSWs (Ohala, 1983). In the voiced/voiceless panels, the five Japanese vowels /i/, /e/, /a/, /o/, and /u/ were arranged in order of their articulation locations from the front to the back of the mouth. Consonants characterized by airflow obstruction were arranged from left to right in terms of articulation locations (e.g., bilabial, and alveolar) from the front to the back of the mouth and manners of articulation (e.g., plosive, and fricative).

**Table 2:** Redisplay of syllables significantly related to comfort/discomfort ratings.

**VOICED**									
	**Bilabial**			**Alveolar**				**Palatal**	**Velar**
	**Nasal /m/**	**Plosive /b/**	** 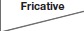 **	**Nasal /n/**	**Affricate /dz/**	**Fricative /z/**	**Affricate /ʤ/**	**Fricative /ʒ/**	**Plosive /g/**
/i/									
/e/		−1.41		−1.19					
/a/					−0.49				
/o/	1.19	−0.56							
/u/									
**VOICELESS**									
	**Bilabial**			**Alveolar**				**Palatal**	**Velar**
	** 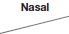 **	**Plosive /p/**	**Fricative /ɸ/**	** 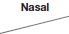 **	**Affricate /ʦ/**	**Fricative /s/**	**Affricate /ʨ/**	**Fricative /ɕ/**	**Plosive /k/**
/i/							−0.87		
/e/		−0.41							
/a/						0.68			
/o/									
/u/		0.63	1.30		0.92	1.13			

*The table primarily classifies syllables into voiced/voiceless. In the voiced/voiceless panels, five Japanese vowels /i/, /e/, /a/, /o/, and /u/ are arranged in order of articulation location (from front to back of the mouth). Consonants are arranged in terms of location and manner of articulation. Comfort (orange)—discomfort (light blue)*.

Table 2 is a display of the syllables that were significantly different from the mean value across the 1,566 cases (0.11) in comfort/discomfort ratings. The first syllables, /m/+/o/, /p/+/u/, /ɸ/+/u/, /ʦ/+/u/, /s/+/a/, and /s/+/u/, were rated as being related to comfort. Their typical sound-symbolic expressions were “moko-moko,” “puni-puni,” “fuwa-fuwa,” “tsuru-tsuru,” “sara-sara,” and “sube-sube.” In the voiced panel, only /m/+/o/ was rated as being related to comfort. On the contrary, in the voiceless panel, the back vowel /u/ was rated as being significantly related to comfort with many consonants (/p/, /ɸ/, /ʦ/, and /s/) and consonant /s/ was also related to comfort regardless of the vowel. The first syllables that were rated as being related to discomfort were /b/+/e/, /b/+/o/, /n/+/e/, /dz/+/a/, /p/+/e/, and /tɕ/+/i/. Their typical sound-symbolic expressions were “beta-beta,” “boko-boko,” “neba-neba,” “zara-zara,” “peta-peta,” and “chiku-chiku.” The front vowels /i/, /e/, and voiced consonants /b/, /n/, and /dz/ were always related to discomfort. In sum, articulation points of vowels could be a dominant factor for comfort/discomfort ratings: positive ratings were associated with the back vowel /u/, accompanied with voiceless consonants, while negative ratings were associated with front vowels, /i/ and /e/.

Tables 3, 4 are displays for bumpy/flat and rough/smooth ratings. The general trends of the tables are similar, and can be characterized in terms of voiced/voiceless. The first syllables, /m/+/o/, /b/+/o/, /dz/+/a/, /dʒ/+/a/, /g/+/a/, /g/+/o/, and /tɕ/+/i/, were related both to bumpy and rough, while /p/+/e/, /ʦ/+/u/, and /s/+/u/ were related both to flat and smooth. Voiced consonants (/m/, /dz/, /dʒ/, and /g/), mainly with vowels /a/ and /o/, were related to bumpy and rough, while voiceless consonants (/p/, /ʦ/, and /s/), mainly with vowels /u/, were related to flat and smooth (/tɕ/+/i/ was an exception).

**Table 3:** Redisplay of syllables related to bumpy/flat ratings.

**VOICED**									
	**Bilabial**			**Alveolar**				**Palatal**	**Velar**
	**Nasal /m/**	**Plosive /b/**	** 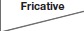 **	**Nasal /n/**	**Affricate /ʣ/**	**Fricative /z/**	**Affricate /ʤ/**	**Fricative /ʒ/**	**Plosive /g/**
/i/									
/e/		−0.96							
/a/					0.87		1.38		1.40
/o/	1.06	1.95							1.33
/u/									0.63
**VOICELESS**									
	**Bilabial**			**Alveolar**				**Palatal**	**Velar**
	** 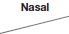 **	**Plosive /p/**	**Fricative /ɸ/**	** 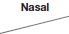 **	**Affricate /ʦ/**	**Fricative /s/**	**Affricate /tɕ/**	**Fricative /ɕ/**	**Plosive /k/**
/i/		−1.00					1.35		
/e/		−1.23							
/a/									
/o/									
/u/					−1.87	−1.67			

*Bumpy (orange)—Flat (light blue)*.

**Table 4:** Redisplay of syllables related to rough/smooth ratings.

**VOICED**									
	**Bilabial**			**Alveolar**				**Palatal**	**Velar**
	**Nasal /m/**	**Plosive /b/**	** 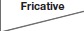 **	**Nasal /n/**	**Affricate /ʣ/**	**Fricative /z/**	**Affricate /ʨ/**	**Fricative /ʒ/**	**Plosive /g/**
/i/									
/e/				−0.88					
/a/					1.52		1.38		1.28
/o/	1.00	1.27							0.92
/u/									
**VOICELESS**									
	**Bilabial**			**Alveolar**				**Palatal**	**Velar**
	** 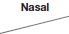 **	**Plosive /p/**	**Fricative /ɸ/**	** 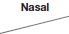 **	**Affricate /ʦ/**	**Fricative /s/**	**Affricate /ʨ/**	**Fricative /ɕ/**	**Plosive /k/**
/i/							2.52		
/e/		−0.64							
/a/						−0.68			
/o/									
/u/		−0.98			−1.70	−1.78			

*Rough (yellow)—smooth (blue)*.

Tables 5, 6 display hard/soft and non-elastic/elastic ratings. The general trends of the tables are similar, and could be characterized in terms of location of articulation (front or back of the mouth). The first syllables, /b/+/o/, /dz/+/a/, /ʦ/+/u/, /g/+/o/, and /k/+/a/, were related both to hard and non-elastic, while /b/+/e/, /p/+/e/, /p/+/u/, /ɸ/+/u/, /n/+/e/, and /g/+/u/, were related both to soft and elastic. The bilabial plosive consonants (/b/, and /p/), bilabial fricative consonant (/ɸ/), and the alveolar nasal consonant (/n/) were related to soft and elastic when combined with the front vowel /e/ or the back vowel /u/ (/b/+/o/ was an exception). Conversely, the alveolar affricate consonants (/dz/, and /ʦ/), and velar plosive consonants (/g/, and /k/) were related to hard and non-elastic feelings (/g/+/u/ was an exception).

**Table 5:** Redisplay of syllables in hard/soft ratings.

**VOICED**									
	**Bilabial**			**Alveolar**				**Palatal**	**Velar**
	**Nasal /m/**	**Plosive /b/**	** 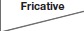 **	**Nasal /n/**	**Affricate /ʣ/**	**Fricative /z/**	**Affricate /ʤ/**	**Fricative /ʒ/**	**Plosive /g/**
/i/									
/e/		−1.50		−1.73					
/a/					0.48		0.96		0.84
/o/		0.87							1.27
/u/									−1.44
**VOICELESS**									
	**Bilabial**			**Alveolar**				**Palatal**	**Velar**
	** 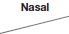 **	**Plosive /p/**	**Fricative /ɸ/**	** 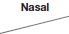 **	**Affricate /ʦ/**	**Fricative /s/**	**Affricate /ʨ/**	**Fricative /ɕ/**	**Plosive /k/**
/i/									
/e/		−1.00							
/a/									1.23
/o/									
/u/		−2.05	−1.73		1.34				

*Hard (orange)—soft (light blue)*.

**Table 6:** Redisplay of syllables in non-elastic/elastic ratings.

**VOICED**									
	**Bilabial**			**Alveolar**				**Palatal**	**Velar**
	**Nasal /m/**	**Plosive /b/**	** 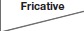 **	**Nasal /n/**	**Affricate /ʣ/**	**Fricative /z/**	**Affricate /ʤ/**	**Fricative /ʒ/**	**Plosive /g/**
/i/									
/e/		−1.00		−1.04					
/a/					0.88				
/o/		0.78							1.00
/u/		−1.33							−1.44
**VOICELESS**									
	**Bilabial**			**Alveolar**				**Palatal**	**Velar**
	** 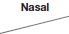 **	**Plosive /p/**	**Fricative /ɸ/**	** 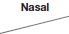 **	**Affricate /ʦ/**	**Fricative /s/**	**Affricate /ʨ/**	**Fricative /ɕ/**	**Plosive /k/**
/i/									
/e/		−0.65							
/a/									0.79
/o/									
/u/		−2.21	−1.16		1.21	0.87			

*Non-elastic (orange)—elastic (light blue)*.

Tables 7, 8 are displays for slippery/sticky and dry/wet ratings. The general trends of the tables are similar, and can also be characterized in terms of location of articulation. The first syllables, /ʦ/+/u/, /s/+/a/, and /k/+/a/, were related both to slippery and dry, while /b/+/e/, /b/+/u/, /p/+/e/, /n/+/e/, and /g/+/u/, were related both to sticky and wet. The bilabial plosive consonants (/b/, and /p/) and alveolar nasal consonant (/n/) were related to sticky and wet when combined with the front vowels /e/, or back vowel /u/. Conversely, alveolar affricate consonants (/ʦ/) and alveolar fricative consonants (/s/), and velar plosive consonant (/k/), were related to slippery and dry (/g/+/u/ was an exception).

**Table 7:** Redisplay of syllables in slippery/sticky ratings.

**VOICED**									
	**Bilabial**			**Alveolar**				**Palatal**	**Velar**
	**Nasal /m/**	**Plosive /b/**	** 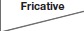 **	**Nasal /n/**	**Affricate /ʣ/**	**Fricative /z/**	**Affricate /ʤ/**	**Fricative /ʒ/**	**Plosive /g/**
/i/									
/e/		−2.29		−2.58					
/a/									
/o/									
/u/		−0.74							−0.66
**VOICELESS**									
	**Bilabial**			**Alveolar**				**Palatal**	**Velar**
	** 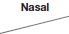 **	**Plosive /p/**	**Fricative /ɸ/**	** 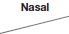 **	**Affricate /ʦ/**	**Fricative /s/**	**Affricate /ʨ/**	**Fricative /ɕ/**	**Plosive /k/**
/i/									
/e/		−1.49							
/a/						1.14			0.98
/o/									
/u/					1.89	1.70			

*Slippery (orange)—sticky (light blue)*.

**Table 8:** Redisplay of syllables in dry/wet ratings.

**VOICED**									
	**Bilabial**			**Alveolar**				**Palatal**	**Velar**
	**Nasal /m/**	**Plosive /b/**	**Fricative**	**Nasal /n/**	**Affricate /ʣ/**	**Fricative /z/**	**Affricate /ʤ/**	**Fricative /ʒ/**	**Plosive /g/**
/i/									
/e/		−1.35		−1.81					
/a/					1.24		1.33		1.00
/o/									1.10
/u/		−1.00							−1.06
**VOICELESS**									
	**Bilabial**			**Alveolar**				**Palatal**	**Velar**
	** 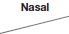 **	**Plosive /p/**	**Fricative /ɸ/**	** 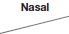 **	**Affricate /ʦ/**	**Fricative /s/**	**Affricate /tɕ/**	**Fricative /ɕ/**	**Plosive /k/**
/i/		−1.91					1.43	−0.89	
/e/		−1.00							
/a/						1.08			1.77
/o/									
/u/		−1.49			1.01				

*Dry (orange)—wet (light blue)*.

Table 9 is a display for warm/cold ratings. Few specific sounds were related to warm/cold ratings. The bilabial nasal /m/ and bilabial fricative /ɸ/ were found to be related to warm when combined with the back vowel /o/ and /u/, respectively. The bilabial plosive /p/ and alveolar affricate /ʦ/ were related to cold when combined with the front vowel /i/ and back vowel /u/, respectively.

**Table 9:** Redisplay of syllables in warm/cold ratings.

**VOICED**									
	**Bilabial**			**Alveolar**				**Palatal**	**Velar**
	**Nasal /m/**	**Plosive /b/**	** 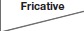 **	**Nasal /n/**	**Affricate /dz/**	**Fricative /z/**	**Affricate /ʤ/**	**Fricative /ʒ/**	**Plosive /g/**
/i/									
/e/									
/a/									
/o/	1.00								
/u/									
**VOICELESS**									
	**Bilabial**			**Alveolar**				**Palatal**	**Velar**
	** 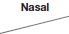 **	**Plosive /p/**	**Fricative /ɸ/**	** 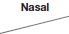 **	**Affricate /ʦ/**	**Fricative /s/**	**Affricate /ʨ/**	**Fricative /ɕ/**	**Plosive /g/**
/i/		−1.82							
/e/									
/a/									
/o/									
/u/			0.91		−1.18				

*Warm (orange)—cold (light blue)*.

## Discussion

Although most research on sound symbolism has focused on cross-modal associations of speech sounds with visual shapes (e.g., Köhler, 1929; Ramachandran and Hubbard, 2001) and gustation (e.g., Gallace et al., 2011; Sakamoto and Watanabe, 2016), sound symbolism related to tactile perceptual qualities has received little attention. In this study, we demonstrated the existence of sound symbolism in touch in a comprehensive manner by using spontaneously produced sounds of Japanese SSWs. Specifically, evaluations of tactile fundamental properties could be symbolized by spontaneously produced first syllables of Japanese SSWs expressing subjective experiences when touching material samples.

Our analysis of associations between the first syllables and tactile qualities revealed that for vowels, positive tactile impressions were associated with the back vowel /u/, and negative tactile impressions were associated with the front vowels /i/ and /e/. Central vowels (/o/ and /a/) were mainly associated with rough, hard, and dry feelings. For consonants, voiced consonants (e.g., /dz/ and /g/) were associated with feelings of roughness, and voiceless consonants (e.g., /ʦ/, and /s/) were associated with feelings of smoothness. Bilabial plosives (/p/ and /b/) and the voiced alveolar nasal /n/ consonant were primarily associated with soft, elastic, sticky and wet feelings. Conversely, the alveolar affricate consonants /dz/ and /ʦ/, and velar plosive consonants /k/ and /g/ were mainly associated with hard and non-elastic feelings. The voiceless alveolar affricate consonant /ʦ/, voiceless alveolar fricative consonant /s/, and voiceless velar plosive consonant /k/ were associated with slippery and dry feelings.

Tactile modality is considered to play an important role in evaluating objects in daily life, such as product design (e.g., Peck and Childers, 2003; Spence and Gallace, 2011), clothing (e.g., Workman, 2010), and cosmetics (e.g., Nakatani et al., 2013). Furthermore, tactile perceptual dimensions have been studied in a large body of psychophysics literature (e.g., Yoshida, 1968; Hollins et al., 1993; Gescheider et al., 2005; Bergmann Tiest and Kappers, 2006; Chen et al., 2009) Most psychophysical studies on the perceptual space of touch have used the multidimensional scaling method and/or the semantic differential method. However, analyzing the sensory vocabulary of touch could be an alternative and effective way of investigating categories of tactile perception (see also Guest et al., 2011; Dingemanse and Majid, 2012; Doizaki et al., 2017). In this study, we investigated associations between syllables of Japanese sound-symbolic expressions and fundamental tactile perceptual qualities. Our detailed observations of the associations might provide a clue to obtaining a comprehensive picture of tactile perceptual categories. For example, from the articulation location of the syllables in Table 1, tactile comfort feelings could be classified into two or three categories: smooth and slippery comfort (/ʦ/+/u/, /s/+/u/, and /s/+/a/), warm comfort (/ɸ/+/u/, and /m/+/o/), and elastic comfort (/p/+/u/). This classification might be related to the articulation of alveolar (/ʦ/, and /s/) and bilabial (/ɸ/, /m/, and /p/) sounds. Conversely, discomfort feelings could be classified into two categories: rough discomfort (/dz/+/a/, /b/+/o/, and /tɕ/+/i/), and sticky discomfort (/b/+/e/, /p/+/e/, and /n/+/e/).

From here on, we discuss tactile perceptual categories based on the sound symbolism obtained in our experiment. A previous review (Okamoto et al., 2013) suggested that fundamental dimensions of tactile perceptual space were rough/smooth, hard/soft, slippery/sticky, and warm/cold, and therefore the number of possible tactile categories could be all of their combinations. However, in reality, smooth property can be accompanied with a hard-slippery property or soft-sticky property. The first syllables /ʦ/+/u/ and /s/+/u/ (the typical SSWs “tsuru-tsuru,” and “sube-sube” were used for materials such as glass) were related to flat, smooth, hard, non-elastic, slippery and dry evaluations. Conversely, the first syllables /b/+/e/, /n/+/e/, and /p/+/e/ (the typical SSWs “beto-beto,” “neba-neba,” and “peta-peta” were used for materials such as slime), were related to flat, smooth, soft, elastic, sticky, and wet evaluations. Roughness property can be accompanied with hard sensation. The first syllables, /b/+/o/, /dz/+/a/, and /g/+/o/ (the typical SSWs “boko-boko,” “zara-zara,” and “gotsu-gotsu” were used for materials such as sandpaper), were related to bumpy, rough, hard, non-elastic, and dry evaluations. Hierarchical cluster analysis also suggested the categorization of fundamental tactile qualities (Figure 1). In general, the first–third cluster correspond to the comfort tactile category and the forth–sixth cluster correspond to the discomfort tactile category. This implies that comfort/discomfort is a critical criterion for tactile categorization, which is in agreement with the findings of our previous study using the semantic differential method (Sakamoto and Watanabe, 2017).

We analyzed sound symbolism in terms of voiced/voiceless, locations of articulation, and manners of articulation. Our results suggested that articulation location of vowels could be a determining factor for comfort/discomfort ratings (back vowel for comfort, and front vowels for discomfort). We showed that /ʦ/+/u/, /s/+/u/, /s/+/a/, /ɸ/+/u/, /m/+/o/, and /p/+/u/ were associated with comfort, while /b/+/o/, /dz/+/a/, /tɕ/+/i/, /b/+/e/, /n/+/e/, and /p/+/e/ were associated with discomfort. These results are interestingly similar to the results of that of gustation. Sakamoto and Watanabe (2016) showed that /s/, /h/ (/ɸ/ in the current study), /a/, and /sy/ (/ɕ/ in the current study) are related to good taste and texture, and that consonants /g/, /b/, /z/ (/dz/ in the current study), /i/, and /d/ are related to bad taste and texture. These common trends are consistent with the idea proposed by Ohala (1983) and Hinton et al. (1994) that voiced consonants (e.g., /g/, /b/, and /n/) and front vowels (e.g., /i/, and /e/) articulated by closing the mouth are related to negative emotion because such phonemes require pressure when articulating utterances. Conversely, clear speech sounds, such as /s/, /ɸ/, and the vowel /a/, are articulated by opening one's mouth and are related to positive emotion because of a lack of pressure in articulating utterances.

For other tactile perceptual qualities, sound symbolism for bumpy/flat and rough/smooth ratings were related to voice/voiceless (i.e., the only difference being vibration of the vocal cords). Roughness quality was found to be related to voiced consonants, and smoothness quality to voiceless consonants. As for sound symbolism for hard/soft, non-elastic/elastic, slippery/sticky and dry/wet, location of articulation was also identified as a possible factor. The bilabial and alveolar nasal consonants were always related to soft, elastic, sticky and wet. The bilabial and nasal consonants are generated by stopping the airflow with the upper and lower lips and by redirecting it through the nose, respectively. In other words, the lip and nose (i.e., soft tissues of the human body) are used to generate bilabial and nasal consonants. Conversely, the alveolar affricate, alveolar affricate, and velar plosive consonants were found to be associated with hard, non-elastic, slippery and dry feelings. These consonants are produced close to the superior alveolar ridge (relatively hard portion). Gick and Derrick (2009) demonstrated that the event-relevant tactile information (air puffs were applied during speech perception) could influence speech perception, and that this action occurs without previous training. In addition, Lari Vainio and colleagues (e.g., Vainio et al., 2013, 2016) highlighted a link between grip and articulation that could be related to the mechanism of articulation mimicking tactile experiences. Taking the interaction between tactile sensation and perception/action of speech into account, the underlying mechanism of sound symbolism in these features might be related to how the sound is pronounced (vibration of the vocal cords, and tactile feature of articulation locations). The relationship among speech sounds, articulation, and touch might also be related to the “mouth gesture” hypothesis proposed by Paget (1930) that the mouth and other articulators often echo hand movements. Human beings unintentionally move mouth-imitating attributes of the referents of words. Speech sounds arise in the process of the mouth, tongue, and lips involuntarily imitating body movements (Hawhee, 2006).

In Tables 2–9, we also examined manner of articulation (plosive, affricate, and fricative). Plosive and fricative consonants are generated by interrupting airflow with a complete closure and with a narrow constriction before releasing the airflow, respectively. Affricates lie in between plosives and fricatives, beginning with complete obstruction of the airflow (plosive) and ending with incomplete closure (fricative). Non-words containing fricative consonants have been reported as being perceived as smaller, faster, lighter, sharper, softer, and more feminine than plosives (Klink, 2000). In our tactile study, the bilabial plosive consonants (/b/, and /p/) were related to soft, elastic, sticky, and wet feelings, while velar plosive consonants (/k/, and /g/) were associated with bumpy, rough, hard, non-elastic, and dry feelings. The alveolar fricative consonant /s/ was related to comfort, flat, smooth, non-elastic, slippery, and dry feelings. We could not find a consistent trend for the two types of plosives. However, the bilabial plosive and alveolar fricative might be a pair of sounds that is related to the opposite tactile qualities. In terms of analyzing acoustic features underlying sound symbolism, Knöferle et al. (2017) highlighted different acoustic cues underlying sound-size and sound-shape mappings. Specifically, F1 in combination with F2 and duration are related to size symbolism, while shape symbolism is related to F2 and F3. For example, larger size tends to be mostly expressed by using vowels with high F1, low F2, and longer duration. Conversely, visual roundness tends to be mostly conveyed by vowels that are low in F2 and high in F3.

Although we have shown the systematic association between sound and touch in Japanese, the generality/variety between languages is an issue awaiting further investigation. Sound symbolism has been observed in languages worldwide. Even English has SSWs not only for visual sensations, but also for tactile ones, although there are few. Words, for example, starting with “sl-,” such as slime, slush, slop, slobber, slip, and slide are associated with something smooth or wet (Bloomfield, 1933). Whether the similar analysis to the current study can be applied to Indo-European languages is an interesting direction. On the contrary, although previous research has attempted to grasp the fundamental factors of human tactile perception and universal perceptual space of touch, perceptual space may vary depending on the language and interests or goals of the perceivers. In fact, recent works have shown East/West cross-cultural differences in the bouba-kiki effect (Chen et al., 2016). For possible future research, therefore, if we obtained cross-cultural data by following our proposed method, we could find fundamental tactile factors and culturally variant perceptual space that might act as a deferent among cultures and languages.

## Author contributions

MS and JW conceived the experiments. MS performed the experiment and JW carried out the data analyses. All authors discussed and interpreted the results, and contributed to drafts of this paper.

### Conflict of interest statement

JW is employed by NTT Communication Science Laboratories, Nippon Telegraph and Telephone Corporation as a research scientist conducting basic scientific research on human sensory processing. There are no patents, products in development or marketed products to declare. This does not alter the authors' adherence to the journal's policies on sharing data and materials. The other author declares that the research was conducted in the absence of any commercial or financial relationships that could be construed as a potential conflict of interest.
